# Microwave-assisted intralesional curettage combined with other adjuvant methods for treatment of Campanacci III giant cell tumor of bone in distal radius: a multicenter clinical study

**DOI:** 10.3389/fonc.2024.1383247

**Published:** 2024-05-03

**Authors:** Haocheng Cui, Jianhua Li, Kai Zheng, Ming Xu, Guochuan Zhang, Yongcheng Hu, Xiuchun Yu

**Affiliations:** ^1^ Orthopedic Department, 960 Hospital of People’s Liberation Army, Jinan, Shandong, China; ^2^ Department of Musculoskeletal Tumor, The Third Hospital of Hebei Medical University, Shijiazhuang, Hebei, China; ^3^ Department of Bone Oncology, Tianjin Hospital, Tianjin, China

**Keywords:** giant cell tumor, microwave ablation, distal radius, autologous fibular reconstruction, comparative study

## Abstract

**Objective:**

To compare the clinical outcomes of microwave-assisted intralesional curettage(MAIC) with those of *en bloc* resection and autogenous fibular reconstruction (EBR-AFR) for treating grade III giant cell tumor of the bone (GCTB) of the distal radius and to elucidate the indications for wrist preservation surgery.

**Materials and methods:**

In this retrospective study, 19 patients with grade III GCTB of the distal radius who underwent surgery at three medical institutions were included and categorized based on their surgical pattern. Seven patients underwent MAIC and internal fixation with bone cement (MAIC group) and 12 underwent EBR-AFR (EBR-AFR group). To evaluate the function of the affected limb postoperatively, wrist range of motion, grip strength, Musculoskeletal Tumor Society (MSTS) scores were recorded.

**Results:**

The follow-up time of the MAIC group was 73.57 ± 28.61 (36–116) months, with no recurrence or lung metastasis. In contrast, the follow-up time of the EBR-AFR group was 55.67 ± 28.74 (36–132) months, with 1 case of local recurrence (8.3%, 1/12) and 1 case of lung metastasis (8.3%, 1/12). The wrist flexion, extension, supination, pronation, grip strength were better in the MAIC group than in the EBR-AFR group. Although there was no statistically significant difference in the MSTS score between the two groups, it is noteworthy that the MAIC group exhibited significantly superior emotional acceptance and hand positioning compared to the EBR-AFR group(*p* < 0.05).

**Conclusion:**

The functional outcomes of the MAIC group are better. The treatment strategy for grade III GCTB of the distal radius should be determined based on the specific preoperative imaging findings. Nevertheless, MAIC can be the preferred surgical approach for most patients with grade III GCTB of the distal radius, particularly for young patients.

## Introduction

A giant cell tumor of the bone (GCTB) is a locally invasive type of primary bone tumor with a susceptibility age of 20–40 years; this tumor is slightly more prevalent in women. In Western countries, GCTB accounts for approximately 3%–8% of all primary bone tumors; in contrast, it is more common in Asia, particularly in China, accounting for 11.61%–16.7% of all primary bone tumors (1). Except for the distal femur and proximal tibia, the distal radius is the most common site of GCTB (2). This GCTB site is distinct owing to its more aggressive behavior, with a stronger recurrence tendency and risk of lung metastasis (3–6).

The primary goal of treatment strategies for GCTB of the distal radius is to completely resect the tumor and preserve the wrist. Curettage or en bloc resection of the lesion with subsequent reconstruction is the common treatment modality. To achieve an adequate surgical margin, en bloc resection, except for intralesional curettage, is commonly performed as a standard surgery for aggressive lesions in the distal radius (7). Although the local recurrence rate of en bloc resection is low, this surgical approach with larger trauma and complex reconstruction methods may result in more complications (5, 7). Furthermore, with an increasing demand for wrist function among patients, particularly young patients, a less destructive option is preferred (8). Intralesional curettage can preserve the wrist joint, is relatively simple, has a low complication rate, and can obtain a higher wrist function score; however, the risk of incomplete tumor resection and high recurrence rate remains (9, 10).

How to decrease the local recurrence rate of GCTB while preserving wrist joint function? Many adjuvant therapies, including alcohol, liquid nitrogen, hydrogen peroxide, electrocautery, and bone cement, have been applied for clinically treating GCTB (11–13). These adjuvant therapies combined with intralesional curettage have achieved clinical results in decreasing recurrence and improving limb function postoperatively.

Since the 1980s, microwave ablation has been employed in clinical settings. At present, it is widely utilized in the adjuvant treatment of various tumors, including bone tumors, and forms a clinical standard for treating bone tumors using microwave ablation technology (14). In contrast to other adjuvant therapies administered post-curettage, microwave inactivation serves as a preoperative adjuvant therapy that effectively mitigates intraoperative blood loss and minimizes the risk of tumor contamination in surrounding tissues (15). In the present study, to elucidate the feasibility of curettage with microwave ablation for treating GCTB of the distal radius, we retrospectively analyzed 19 patients who underwent different surgical methods at three hospitals. The two major study objectives were as follows: (1) comparing the efficacy of two surgical methods [microwave-assisted intralesional curettage(MAIC) and en bloc resection and autogenous non-vascularized fibular reconstruction (EBR-AFR)] for treating GCTB of the distal radius; and (2) elucidating the surgical indications for microwave-assisted curettage in patients with Campanacci III distal radius GCTB.

## Patients and methods

### Patients

The medical records of patients who underwent surgery between 2005 and 2019 at three musculoskeletal oncology centers in China (PLA 960^th^ Hospital, The Third Hospital of Hebei Medical University, and Tianjin Hospital) were retrospectively reviewed. The Ethics Committee of PLA 960^th^ Hospital approved this study. Informed consent was obtained from all participants.

The inclusion criteria were as follows: (i) patients with Campanacci III GCTB of the distal radius and a follow-up time of >36 months; (ii) patients who underwent MAIC or EBR-AFR; and (iii) the primary evaluation indicators included oncological and functional outcomes of the wrist. The exclusion criteria were as follows: (i) patients with Campanacci I or II GCTB and a follow-up time of <36 months; (ii) patients who did not undergo MAIC or EBR-AFR; and (iii) patients with incomplete clinical data.

Based on the surgical method, the patients were divided into two groups: the MAIC and EBR-AFR groups. The patients in both groups were rechecked every 3 months in the first year postoperatively and every 6 months from the second year postoperatively. The surgical site and chest were subjected to routine imaging examinations [X-ray and computed tomography (CT), with or without magnetic resonance imaging (MRI)], and the surgical complications were simultaneously recorded.

All patients underwent clinical and radiographic assessments. Wrist function was evaluated using the revised functional evaluation system of the Musculoskeletal Tumor Society (MSTS) (16). MSTS score encompasses pain, functional movement evaluation, subjective emotional acceptance, hand positioning analysis, hand flexibility examination, and upper limb lifting capacity assessment. Each of these components can be rated on a scale from 0 to 5, with the cumulative scores yielding a total score of 30. Furthermore, a dynamometer was used to measure grip strength, and a goniometer was used to measure the range of motion (ROM) of the wrist and compare it with the contralateral side. Radiographs of the operated forearm and wrist were obtained and used to study radiographic bone union, tumor recurrence, and subluxation of the radiocarpal joint and distal radioulnar joint (DRUJ).

The clinical data and functional indexes of both groups were compared at 36 months post-surgery. Data are expressed as mean ± standard deviation, percentage, or the number of patients in each group. The student’s t-test was used to analyze the quantitative data, whereas the Fisher’s exact test was used to analyze the qualitative data. SAS statistics software (Version 9.13) was used to perform the analysis. A *p*-value of <0.05 was considered statistically significant.

### Surgical procedures

#### MAIC

The surgical procedure was similar to the conventional curettage procedure; however, before curettage surgery, a soft tissue extension should be exposed. A gauze was used to isolate the soft tissue extension, tumor bone, and surrounding normal tissues. Then, a microwave antenna was evenly inserted into the tumor segment for ablation. An ECO system (2450 MHz, ECO-100A1, YiGAO, Nanjing, China) was used to perform microwave ablation.

The shaft position of the microwave antenna was repositioned between the ablation cycles to obtain a larger thermoablation zone and positioned at the lesion edge to achieve a larger ablation margin by controlling the power (confirmed via temperature measurements). The ablation range was more than 2 cm beyond the boundary of the tumor tissue (17). On the articular side, if the subchondral bone was less than 1 cm, the abovementioned requirements need not be met. Two syringes were simultaneously inserted into the joint cavity of the wrist and continuously monitored using thermometry needles. Cryogenic saline was used to cool the articular cavity to protect the normal structure. Furthermore, cooled sterilized water was applied to ensure that the temperature of adjacent normal tissues was <43°C.

After completing the ablation process, the cortical bone window was expanded, and the fully inactivated tumor tissue was completely scraped with a curette. Then, the four walls of the tumor cavity were further burned with an electric knife to remove any potential residual tumor tissues. After washing the cavity with large amounts of normal saline, it was filled with an appropriate amount of bone cement. Internal fixation was performed using a distal radius plate.

#### EBR-AFR

The surgical approach was selected based on the location of the tumor. Long incisions were made on the dorsal side of the radius or the palmar side. Then, the interosseous membrane was opened, the wrist joint was flexed, and blood vessels and nerves were protected. The flexor and extensor tendons were separated and distal radius osteotomy was performed at least 3 cm outside the tumor boundary based on preoperative MRI tumor boundary planning. Then, the pronator muscle and tumor mass were removed.

Based on the length of the radius defect, an appropriate length of the proximal fibula was measured and cut, ensuring that the common peroneal nerve was protected. The fibula segment was transplanted to the radius defect and connected with a radius stump with a compression plate. The fibula tip was maintained in the same direction as the original styloid process of the radius when controlling rotation. A reserved suture was tightly knotted to repair the carpal joint capsule on the palmar side. Then, the dorsal joint capsule was sutured, and the ligament tissue was closely sutured with the residual ligament tissue around the ulna. Thereafter, the wrist joint was reset according to the protocols described by Chadha M (18) and Qi D (19).

The fixation methods were as follows: the first Kirschner wire was allowed to penetrate the transplanted fibular head and the distal end of the ulna to fix and reconstruct the distal radioulnar joint; the second Kirschner wire was allowed to penetrate the transplanted fibular head and scaphoid to fix the “radiocarpal joint”; and the third Kirschner wire was passed from the ulnar head, across the lunate, and into the scaphoid. After the reduced wrist joint was fixed with the Kirschner wire, the plate and screw were tightened again. At 10–12 weeks postoperatively, the Kirschner wire was removed and the affected forearm was fixed with a short splint until the X-ray examination revealed bone healing.

## Results

**Table 1** summarizes the profiles and results of the patients. Nineteen patients with Campanacci III GCTB of the distal radius who underwent surgery at three medical institutions between March 2005 and March 2019 were included in this study. The patients were categorized based on the surgical methods. Seven patients who underwent MAIC and internal fixation with bone cement were included in the MAIC group, with six women and one man. The average age was 40.43 ± 17.54 (22–77) years. There were three cases of the left wrist, four cases of the right wrist, and two cases of pathological fractures. The follow-up time was 73.57 ± 28.61 (36–116) months. On the other hand, 12 patients who underwent EBR-AFR of the wrist joint were included in the EBR-AFR group, with five women and seven men. The average age was 31.33 ± 11.64 (16–59 years). There were eight cases of the left wrist, four cases of the right wrist, and four cases of pathological fractures. The average follow-up time was 55.67 ± 28.74 (36–132) months.

**Table 1:** Baseline characteristics of the enrolled cases.

	MAIC group	EBR-AFR group	Statistical value
Patients (n)	7	12	
Age, years (average± SD)	40.43 ± 17.54	31.33 ± 11.64	t=1.37 P=0.189
Gender(M/F)	1/6	7/5	P=0.147
Side(left/right)	3/4	8/4	P=0.377
Pathological fracture (n)	2	4	
Time of follow up,months (average± SD)	73.57 ± 28.61	55.67 ± 28.74	t=1.31 P=0.207

F, Female; M, male.

### MAIC group

The surgery was successfully completed without intraoperative complications, and primary wound healing was achieved in all patients. The average follow-up time was 73.57 ± 28.61 (36–116) months. One case of flexor tendon injury caused by trauma 2 months postoperatively was repaired. To date, the patient has been followed up for 83 months, with good recovery of wrist joint function.

**Figure 1:**
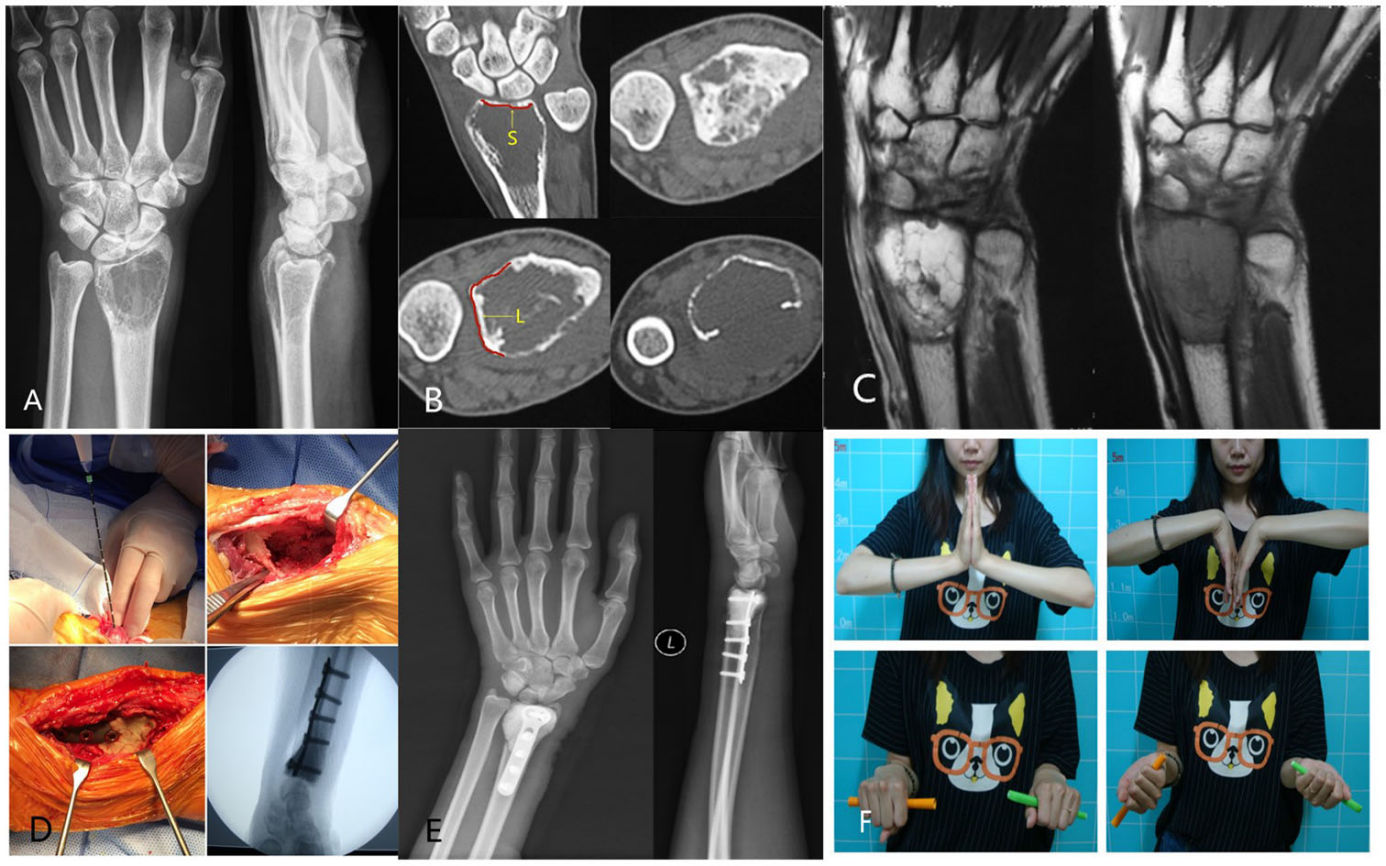
**(A)** Preoperative radiographs displayed expansive bone destruction at the distal end of the left radius with discontinuous bone cortex. **(B)** Preoperative CT showed cortical perforation on the palmar side of the radius, soft tissue extension, and no involvement of the radiocarpal joint surface. The uninvolved radiocarpal joint surface (yellow S).The DRUJ is not affected(yellow L). **(C)** Preoperative MRI evaluation of soft tissue extension can be completely removed. **(D)** Intraoperative microwave ablation combined with curettage and internal fixation with bone cement were used for treatment. **(E)** After 36 months of follow-up, the X-ray film showed that the internal fixation was in good position, and there was no local recurrence. **(F)** After 36 months of follow-up, wrist mobility was good.

In the MAIC group, the average ROM of the wrist was as follows: 65.71° ± 3.45° of extension, 47.57° ± 6.35° of flexion, 67.71° ± 3.82° of supination, 77.29° ± 4.03° of pronation, 17° ± 1.41° of radial deviation, and 25.86° ± 3.29° of ulnar deviation. The average percentage of grip strength was 83.29% ± 5.96% compared with that of the contralateral side. The average MSTS score was 26.71 ± 1.89. A typical case: a 32-year-old woman presented with a painful mass on the left wrist for 6 months that aggravated for 2 weeks. She was diagnosed with GCTB of the distal left radius (Campanacci III) and underwent MAIC and internal fixation with bone cement filling. After 36 months of follow-up, her recovery was good, with no local recurrence and lung metastasis (**Figure 1**).

### EBR-AFR group

In the EBR-AFR group, 11 patients were treated using the dorsal approach and 1 patient was treated using the volar approach. The average length of fibula resection was 6.4 ± 1.0 (5–8) cm. The surgery was successfully completed in all patients, with no intraoperative complications. Furthermore, all incisions healed in one stage postoperatively. All patients underwent fibular transplantation and bone healing within 6–12 months postoperatively, with no common peroneal nerve paralysis or ligament dysfunction associated with the donor site. The average follow-up time was 55.67 ± 28.74 (36–132). Local recurrence was observed in one patient at 28 months postoperatively; a soft tissue mass was formed. After secondary resection of the mass and reconstruction of the partially damaged extensor pollicis longus tendon via extensor tendon transplantation, the patient was followed up for 39 months without recurrence. Lung metastasis was observed in one patient at 32 months postoperatively. The pulmonary nodules were stable after 52 months of follow-up after gamma knife radiotherapy.

At the last follow-up, imaging examinations revealed radiographic signs of radiocarpal degenerative arthritis in 11 patients based on X-ray findings. According to the scoring system reported by Haus (20), there was one patient with grade 0, three with grade 1, four with grade 2, and four with grade 3 arthritis. Additionally, 10 patients exhibited varying degrees of bone resorption, including six patients with DRUJ separation, seven patients with wrist ulnar deviation, two patients with carpal volar subluxation, one patient with dorsal subluxation, and one patient with dorsal complete dislocation.

The MSTS score averaged 24.47 ± 2.55. The average ROM of the wrist was as follows: 35.83° ± 14.52° of extension, 14.00° ± 8.36° of flexion, 44.08° ± 24.78° of supination, 57.25° ± 18.94° of pronation, 15.50° ± 6.71° of radial deviation, and 19.42° ± 10.14° of ulnar deviation. Grip strength averaged 55.25% ± 29.38% compared with the contralateral side.

**Figure 2:**
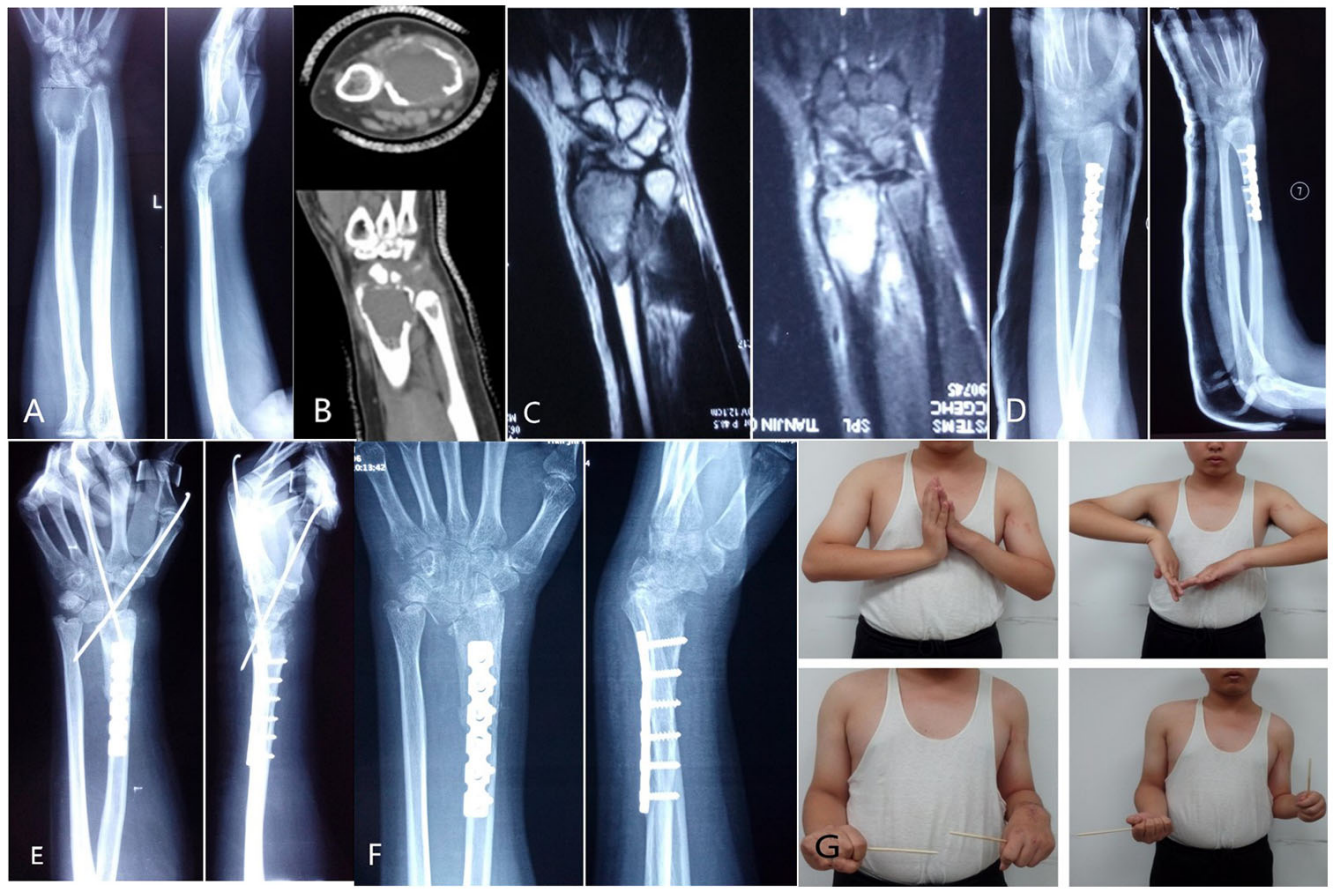
**(A)** The first preoperative X-ray film showed expansive bone destruction at the distal end of the left radius, discontinuous bone cortex, and volar subluxation of the radiocarpal joint. **(B)** The first preoperative CT showed that the metacarpal and dorsal bone cortex of the distal radius were perforated and the articular surface was involved. **(C)** The first preoperative MRI showed that the tumor invaded the ulnar soft tissue with unclear boundary. **(D)** After resection of tumor segment and reconstruction of wrist joint with autologous fibular head transplantation, X-ray showed that the fracture line was clear and the broken end was well aligned, and the wrist joint was fixed with external fixation brace. **(E)** Local recurrence and dorsal dislocation of wrist joint 2 years after operation. Secondary tumor resection, partial resection of extensor pollicis longus tendon, transposition and repair of extensor digitorum tendon, wrist reduction and Kirschner wire fixation. **(F)** The X-ray image 39 months after surgery showed radiocarpal arthritis (grade 3), distal radioulnar joint separation, wrist ulnar deviation and dorsal dislocation. **(G)** The patient was followed up for 39 months and showed poor wrist flexion, extension and supination.

A typical case: A 16-year-old male presented with recurrent GCTB of the left distal radius 2 years after initial surgery. Diagnosis confirmed recurrent GCTB of the left distal radius. The patient underwent excision of the tumor segment and autologous fibula transplantation to reconstruct the wrist joint. At 2 postoperative years, the patient exhibited radial mass and dislocation of the radiocarpal joint. Subsequently, the patient was readmitted to the hospital for tumor resection and Kirschner wire wrist fixation. After a 39-month follow-up period, the patient demonstrated satisfactory recovery, with no evidence of local recurrence or lung metastasis (**Figure 2**).

### Comparative analysis of the group indices

Statistical comparative analysis revealed no significant differences in age, sex, follow-up time, ulnar deviation, radial deviation, or MSTS score between the two groups (*p* > 0.05). However, extension, flexion, supination, pronation and grip strength were significantly improved in the MAIC group compared to the EBR-AFR group (*p* < 0.05). Furthermore, a comparison was conducted on the six indicators of the MSTS score, revealing that emotional acceptance and hand position function in the MAIC group exhibited significantly superior performance compared to those in the EBR-AFR group (p < 0.05) (**Table 2**). Further analysis of MSTS scores in the two groups at different follow-up time points after surgery revealed a continuous improvement in functional scores post-surgery for both groups. Notably, the MAIC group exhibited superior scores compared to the EBR-AFR group at 3rd, 6th, 12th, and 24th months after surgery(*p* < 0.05) (**Table 3**).

**Table 2:** Postoperative functional comparison between the MAIC and EBR-AFR group.

Date	MAIC group	EBR-AFR group	Statistical value
Extension (degrees)	65.71 ± 3.45	35.83 ± 14.52	t=5.30 P=0.001
Flexion (degrees)	47.57 ± 6.35	14.00 ± 8.36	t=9.15 P=0.000
Supination (degrees)	67.71 ± 3.82	44.08 ± 24.78	t=2.48 P=0.024
Pronation (degrees)	77.29 ± 4.03	57.25 ± 18.94	t=2.73 P=0.014
Radial deviation (degrees)	17 ± 1.41	15.50 ± 6.71	t=0.58 P=0.571
Ulnar deviation (degrees)	25.86 ± 3.29	19.42 ± 10.14	t=1.61 P=0.1248
Grip (percent contralateral side)	83.29% ± 5.96%	55.25% ± 29.38%	t=2.47 P=0.0245
MSTS score	26.71 ± 1.89	24.47 ± 2.55	t=2.01 P=0.0601
pain	4.42 ± 0.53	4.35 ± 0.95	t=0.18 P=0.8608
function	4.42 ± 0.53	4.25 ± 0.45	t=0.75 P=0.4664
emotional acceptance	4.57 ± 0.53	3.81 ± 0.49	t=3.17 P=0.0056
hand positioning	4.57 ± 0.78	3.56 ± 0.83	t=2.61 P=0.0182
dexterity	4.86 ± 0.38	4.75 ± 0.45	t=0.54 P=0.5947
lifting ability	3.86 ± 0.69	3.75 ± 0.87	t=0.29 P=0.7790
Local Recurrence	0%	8.3%(1/12)	
Lung Metastasis	0%	8.3%(1/12)	

MSTS, Musculoskeletal Tumor Society.

**Table 3:** Comparison of postoperative MSTS functional scores between two cohorts of patients with Campanacci III distal radial GCTB.

	Patients (n)	3 months PO	6 months PO	12 months PO	24 months PO	36 months PO
MAIC group	7	20.85 ± 1.34	22.86 ± 1.21	25.86 ± 1.77	26.42 ± 1.13	26.86 ± 1.68
EBR-AFR group	12	19.08 ± 1.89	20.08 ± 1.92	24.17 ± 1.26	24.33 ± 1.61	25.25 ± 2.38
Statistical value		t=2.17	t=3.43	t=2.43	t=3.01	t=1.57
		P=0.0446	P=0.0032	P=0.0263	P=0.0078	P=0.1353

PO, post-operative.

## Discussion

### Surgical selection of Campanacci III GCTB of the distal radius

GCTB of the distal radius exhibits a higher recurrence rate compared to other limb bones (2). This may be attributed to the complex anatomical features of the distal radius, incomplete exposure, and resection during surgery, resulting in residual tumor tissue in potential locations (21). Currently, the preferred treatment option for Campanacci I and II GCTB of the distal radius is intralesional curettage (22). Nevertheless, the management of Campanacci III distal radial GCTB remains a subject of substantial controversy (5, 6, 23).

The controversy arises from the frequent occurrence of soft tissue extension in Campanacci III GCTB of the distal radius, which indicates a high degree of tumor invasion. Li et al. (24) suggested that soft tissue extension in GCTB is an independent risk factor for local tumor recurrence. Zou et al. (6) proposed that tumor size (diameter ≥ 5 cm) and the presence of soft tissue extension are independent risk factors for recurrent GCTB of the distal radius, whereas fractures and surgical approach (intralesional curettage or en bloc resection) are not associated with recurrence risk.

Thus, managing soft tissue extension is crucial for preventing local tumor recurrence. Some experts even consider it as an indication for en bloc surgery (25), recommending en bloc resection as the standard surgical approach for grade III GCTB of the distal radius. This method can reduce the risk of local recurrence and achieve a safe surgical margin (26).

Numerous reconstruction methods are available following en bloc resection, including total wrist arthrodesis, partial wrist arthrodesis, fibular transplantation (18), allograft bone transplantation with an articular surface (27), and customized artificial joint prostheses (28). The reconstruction methods can be categorized into two primary classifications: arthrodesis and arthroplasty. However, both of these reconstruction methods pose a significant challenge for the osteo-oncologist due to their inherent advantages and disadvantages.

Total wrist arthrodesis effectively stabilizes the wrist joint without causing pain and mitigates potential complications associated with joint replacement, including subluxation, dislocation, and degeneration. Furthermore, it alleviates the accompanying pain caused by these complications. Qu et al. (10) conducted total wrist fusion in 8 patients utilizing autogenous fibular grafts as reconstruction bridging materials, resulting in enhanced grip function compared to arthroplasty.

Therefore, this reconstruction technique is typically employed for patients necessitating extensive physical exertion. Moreover, total wrist fusion can serve as a viable alternative in cases where arthroplasty proves ineffective.

While total wrist arthrodesis can offer a stable and robust wrist, the restricted range of motion in the wrist may impede patients’ ability to perform certain daily activities. Partial wrist arthrodesis contributes to the preservation of the metacarpal joint, thereby enhancing the overall quality of life. There are three distinct types of partial wrist fusion, namely radius-lunate fusion, radius-scaphoid fusion, and radius-scaphoid-lunate fusion (29). Notably, among these options, the biomechanical impact of radius-scaphoid-lunate fusion closely resembles that observed in a healthy wrist. Zhu et al. (30) conducted a comparative analysis of functional and radiographic outcomes between partial wrist fusion (fibula-scaphoid-lunate fusion) and autologous fibular graft-based wrist reconstruction. They found that partial wrist fusion resulted in a stable wrist, acceptable motion, good long-term function, and low incidence of complications. In terms of wrist flexion and extension function, arthroplasty demonstrates superior outcomes compared to partial joint fusion. Conversely, partial joint fusion exhibits greater grip strength than arthroplasty.

However, a significant challenge associated with partial wrist fusion is the limited extent of fusion contact. Therefore, long-term stable fixation is required to achieve bone healing. Partial wrist fusion may also lead to complications such as infection, fracture, delayed union, and nonunion. Despite minimal disparity in grip strength, a considerable number of patients remain ineligible for wrist fusion surgery due to compromised range of motion in the wrist joint (31).

The primary advantage of wrist arthroplasty over wrist fusion lies in its capacity to offer enhanced flexibility in joint movement, thereby potentially enhancing patients’ overall quality of life. Due to the anatomical similarity between the distal radius and the proximal fibula, autologous fibular head transplantation is preferred. In 1979, Pho et al. (32) pioneered the utilization of a vascularized fibular head graft for reconstructing long bone defects following tumor resection in the distal radius.

Since then, this technique (non-vascularized or vascularized autogenous proximal fibular graft reconstruction) has emerged as a prevalent approach for wrist reconstruction following distal radius resection (33). Although numerous studies have reported favorable outcomes in wrist reconstruction utilizing autologous proximal fibula, this technique is closely associated with complications including nonunion, delayed union, bone resorption, and graft-related secondary bone collapse (19, 34). The wrist joint is a complex articulation characterized by intricate anatomical structures and multifaceted functionality, wherein ligaments, articular capsules, the triangular fibrocartilage complex (TFCC), distal interosseous membrane (DIOM), and muscles collectively contribute to upholding the stability of this joint. However, in cases where these crucial components are compromised or excised during tumor resection procedures, it can lead to postoperative instability of the reconstructed joint, resulting in pain, diminished grip strength, and restricted wrist function (35).

Humail S M et al. (34) reported 12 cases of grade III GCTB of the distal radius treated with en bloc resection and fibular transplantation. After a 2-year follow-up, wrist joint function was only 60% of the contralateral side. Saini R et al. (7) reported 12 cases of grade III GCTB of the distal radius treated with en bloc resection and fibular transplantation. After a 5.8-year follow-up, wrist joint grip strength was 71% of the contralateral side. They also reported one case of recurrence, three cases of subluxation, and two cases of bone non-union.

In our study, we observed 12 patients in the EBR-AFR group, with an average follow-up of 55 months. Among them, 11 patients exhibited degenerative arthritis, 10 patients showed varying degrees of bone resorption, six patients had lower radioulnar joint separation, seven patients presented with ulnar deviation, two patients had palmar subluxation, one patient had dorsal subluxation, and one patient had dorsal complete dislocation. The average grip strength was only 55.25% compared to the contralateral side.

Muramatsu et al. (36) recommend arthroplasty for patients with low physical demands and fibula-scapho-lunate (FSL) arthrodesis for younger patients engaged in high-energy activities, aiming to achieve a stable and robust wrist function. In general, the choice of reconstruction method should be based on the patient’s occupation, activity requirements, and wrist stability. Therefore, regardless of the wrist reconstruction method employed following tumor resection, achieving a satisfactory functional outcome for patients with Campanacci III distal radial GCTB remains challenging.

### Clinical efficacy and advantages of microwave ablation-assisted curettage in therapeutic intervention

In contrast to the surgical procedure of en bloc resection and reconstruction, intralesional curettage can maximize the preservation of various wrist joint structures, leading to satisfactory postoperative wrist function. However, its main drawback is the potential for a high local recurrence rate (37), with early literature reports suggesting rates as high as 55% (38).

To address this, many scholars have explored and utilized adjuvant treatment methods to minimize local recurrence following intralesional curettage (39). As early as 1969, Vidal et al. (40) used polymethyl methacrylate (PMMA) as an adjunctive treatment for GCTB, believing that the heat generated during PMMA polymerization could eliminate residual tumor cells.

Several scholars advocate for the rational application of local adjuvant therapy to treat soft tissue extension effectively in grade III GCTB (13, 41). Cheng et al. (42), for instance, extensively removed soft tissue extension during surgery, followed by curettage and local adjuvant therapy to treat residual bone tumors as grade I or II lesions. Similarly, Lackman et al. (13) isolated and dissected soft tissue extension, performed curettage surgery, and then chemically treated the tumor wall with 90% phenol before filling the cavity with PMMA. They reported no difference in local recurrence rates between grade II and III lesions treated with these methods. The local recurrence rate of grade II was 7.7% (2/26) and that of grade III was 5.4% (2/37); furthermore, the overall local recurrence rate was 6.3%.

In recent years, microwave ablation has emerged as a recommended adjunctive treatment for bone tumors, as it maintains joint integrity and achieves biological repair of bone defect lesions (17). Microwave ablation utilizes the thermal effect of microwave electromagnetic fields to inactivate tumors (43), inducing apoptosis, destroying tumor blood vessels, and promoting immunity (44–47). Notably, it differs from other ablation methods by using electromagnetic fields to convert tumor cells into heat sources, starting thermal ablation from within the tumor (48, 49). Experiments have shown that controlling the temperature at 60°C–80°C and maintaining a continuous inactivation time of 30 min during microwave ablation can ensure therapeutic efficacy without compromising cortical bone strength (14).

In clinical settings, microwave ablation achieves tumor inactivation through *in situ* ablation after protecting surrounding normal tissue (50). This maximizes bone tissue continuity, facilitating reconstruction and reshaping of bone tissue, and preventing issues related to osteotomy and bone healing that require consideration during tumor resection, inactivation, and replantation. *In situ* ablation is thus preferred for microwave ablation of limb bone tumors (14).Additionally, the lesions were curetted following microwave ablation, and surgical intervention was downgraded to preserve joint anatomy integrity to the fullest extent possible. Early postoperative rehabilitation exercises facilitated satisfactory functional restoration (51).

Ke J et al. (52) reported on a cohort of 8 patients with distal radius giant cell tumor of bone (all above gradeII) who underwent microwave inactivation-assisted curettage followed by internal fixation with bone cement. The mean follow-up duration was 60.69 ± 29.61 months (range: 24-126 months), and no instances of recurrence were observed. The mean MSTS score at the last follow-up was 28.67 ± 1.63, indicating a satisfactory wrist joint function.

Jiao et al. (53) reported a cohort of 21 patients with distal radius GCTB who had clear pathological results, complete imaging data, and follow-up duration exceeding 18 months; this included 11 cases classified as grade II and 10 cases grade III. Ten patients underwent microwave ablation, followed by lesion curettage and internal fixation with bone cement filling. Additionally, 11 patients underwent segmental resection and reconstruction using non-vascularized autologous proximal fibular graft. After comparison, the microwave group demonstrated significantly superior outcomes compared to the arthroplasty group in terms of operation time, intraoperative blood loss, postoperative wrist dorsiflexion, palmar flexion, and radial deviation. Conversely, the arthroplasty group exhibited better results than the microwave group with regard to postoperative wrist ulnar deviation and MSTS score.

In this study, microwave ablation-assisted curettage was performed on 7 patients with grade III tumors in the MAIC group. During the procedure, precise incisions were made to expose the normal tissues surrounding the tumor, ensuring a margin of 2cm beyond the tumor capsule for effective inactivation of tumor cells. The average duration of follow-up was 73 months, and no instances of local tumor recurrence were observed. The wrist function (extension, flexion, rotation, and pronation) and DASH scores of 7 patients with grade III in the MAIC group exhibited significantly superior outcomes compared to those in the EBR-AFR group.

### Indications for microwave ablation-assisted intralesional curettage in the treatment of grade III GCTB of the distal radius

Microwave ablation proves effective in addressing GCTB with soft tissue extension, initially applied in treating liver tumors via percutaneous puncture (54). The homogeneous origin of soft tissue extension and intraosseous tumor components in GCTB distinguishes them from surrounding normal tissues. By utilizing preoperative MRI to determine tumor tissue extent, precise temperature control of the microwave needle can effectively inactivate soft tissue extension without harming surrounding normal tissues. Microwave ablation serves as an adjunct therapy to enhance tumor resection margin safety (16). Compared to performing curettage surgery after soft tissue mass removal, microwave ablation allows needle insertion for tumor inactivation post soft tissue mass exposure, minimizing the contamination risk of surrounding soft tissues (51).

Based on literature review and clinical observations, rational microwave ablation technology application effectively treats some grade III GCTB of the distal radius, achieving local recurrence control and wrist joint function preservation.

To our knowledge, no summary exists on microwave ablation adjuvant intralesional curettage indications in the treatment of Campanacci III GCTB of the distal radius. Therefore, we propose three treatment indications based on soft tissue extension imaging features:

(i) Soft tissue extension not involving the radiocarpal joint surface and DRUJ of the distal radius;
(ii) Intact cortical bone on both sides of the distal radius, facilitating bone cement filling shaping post-curettage.
(iii) Soft tissue extension minimally invades critical surrounding tendons, blood vessels, and nerves, with the longest diameter of the tumor being less than 5 cm.

The first indication prioritizes the main functional structure of the wrist joint, primarily the radiocarpal joint and DRUJ. The radiocarpal joint transmits wrist axial load and accounts for 40% of flexion function and 66.5% of extension function. The DRUJ, a double fulcrum synovial joint comprising the ulnar notch-radial head and radial head-triangular fibrocartilage, mainly facilitates forearm rotation and mechanical conduction. If preoperative imaging indicates soft tissue extension penetration of the radiocarpal joint or DRUJ, *en bloc* resection, is preferred to mitigate local recurrence risk.

**Figure 3:**
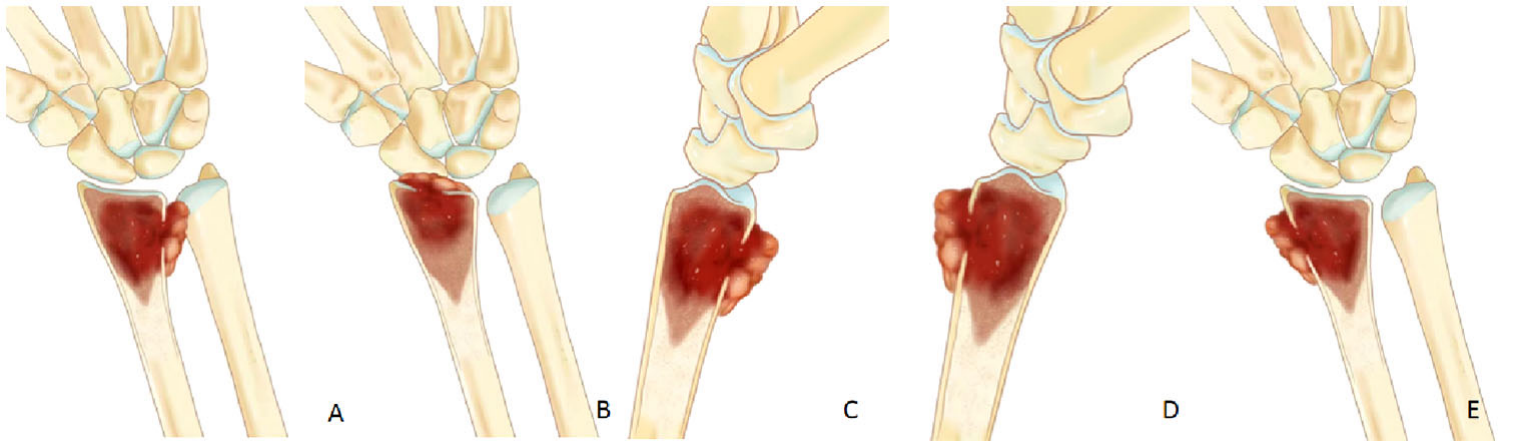
**(A, B)** If soft tissue extension involve the radiocarpal joint or the DRUJ,en bloc should be performed. **(C–E)** If soft tissue extension is located on the palmar, dorsal, or radial side of the distal radius, it can be preserved wrist joint by microwave ablation and curettage.

Conversely, soft tissue extension located on the palmar, dorsal, or radial side of the distal radius, not involving the radiocarpal joint or DRUJ, can be preserved via microwave ablation and curettage, yielding enhanced postoperative function (**Figure 3**).

The second indication pertains to the treatment of the tumor cavity after intralesional curettage, where bone cement is utilized for filling. Bone cement emerges as a recommended choice for tumor cavity repair and reconstruction following GCTB curettage (55). Its advantages include: first, using its thermal effect and cytotoxicity as local adjuvant therapy to eliminate potential residual tumor cells; second, possessing high mechanical strength, offering robust support, and facilitating rapid bone defect reconstruction; lastly, compared to allogeneic bone filling, bone cement filling aids in early tumor recurrence detection, crucial for formulating secondary surgical plans and preserving limb function (56). Thus, as long as at least two intact cortical bones remain in GCTB of the distal radius, cement filling and shaping can be pursued.

The final indication pertains to cases where the GCTB of the distal radius is too large (longest diameter ≥ 5 cm) or has significantly infiltrated surrounding blood vessels, nerves, and other structures. In such instances, wrist joint preservation surgery is not recommended. The primary objective should be complete tumor removal to minimize postoperative recurrence risk.

## Limitations

The present study has some limitations. First, this research had only a small number of patients were included in this study. Second, considering the retrospective nature of this study, as well as its conduction by multiple surgeons across three centers, it is expected that there might have been variations in surgical approaches and specific details; thus, the evidence level is low. Despite these limitations, we assert that microwave-assisted curettage represents a secure and efficacious approach for managing selected Campanacci III GCTB of the distal radius.

## Conclusions

Based on the findings of this retrospective study, we advocate for curettage with adjuvant microwave therapy as a safe and effective approach to treating Campanacci III GCTB of the distal radius. We propose treatment decisions for Campanacci III GCTB of the distal radius should be guided by specific preoperative imaging findings. Microwave ablation-assisted intralesional curettage emerges as a preferred surgical approach for most patients with Campanacci III GCTB of the distal radius, particularly for young patients.

## Data availability statement

The original contributions presented in the study are included in the article/supplementary material. Further inquiries can be directed to the corresponding author.

## Ethics statement

The studies involving humans were approved by Institutional ethical committee of 960 hospital of PLA. The studies were conducted in accordance with the local legislation and institutional requirements. The participants provided their written informed consent to participate in this study. Written informed consent was obtained from the individual(s) for the publication of any potentially identifiable images or data included in this article.

## Author contributions

HC: Data curation, Methodology, Writing – original draft, Writing – review & editing. JL: Data curation, Writing – original draft. KZ: Data curation, Writing – review & editing. MX: Data curation, Investigation, Writing – review & editing. GZ: Data curation, Writing – review & editing. YH: Data curation, Methodology, Writing – review & editing. XY: Investigation, Methodology, Project administration, Writing – original draft, Writing – review & editing.
